# The *ex vivo* pharmacology of HIV-1 antiretrovirals differs between macaques and humans

**DOI:** 10.1016/j.isci.2022.104409

**Published:** 2022-05-16

**Authors:** Carolina Herrera, Mackenzie L. Cottrell, John Prybylski, Angela D.M. Kashuba, Ronald S. Veazey, Javier García-Pérez, Natalia Olejniczak, Clare F. McCoy, Paul Ziprin, Nicola Richardson-Harman, José Alcami, Karl R. Malcolm, Robin J. Shattock

**Affiliations:** 1 Section of Virology, Faculty of Medicine, St. Mary’s Campus, Imperial College London, UK; 2 University of North Carolina at Chapel Hill, UNC Eshelman School of Pharmacy, Division of Pharmacotherapy and Experimental Therapeutics, Chapel Hill, NC, USA; 3 Tulane National Primate Research Center, Tulane University School of Medicine, Covington, LA, USA; 4 AIDS Immunopathology Unit. National Center of Microbiology, Instituto de Salud Carlos III, Madrid, Spain; 5 School of Pharmacy, Medical Biology Centre, Queen’s University of Belfast, Belfast, UK; 6 Department of Surgery and Cancer, St Mary’s Hospital, Imperial College London, UK; 7 Alpha StatConsult, LLC, Damascus, MD, USA; 8 HIV Unit, Hospital Clinic-IDIBAPS, Barcelona, Spain; 9 Centro de Investigación Biomédica en Red de Enfermedades Infecciosas, Instituto de Salud Carlos III (ISCIII), Madrid, Spain

**Keywords:** Natural sciences, Biological sciences, Toxicology, Immunology, Biological sciences research methodologies

## Abstract

Non-human primates (NHP) are widely used for the pre-clinical assessment of antiretrovirals (ARVs) for HIV treatment and prevention. However, the utility of these models is questionable given the differences in ARV pharmacology between humans and macaques. Here, we report a model based on *ex vivo* ARV exposure and the challenge of mucosal tissue explants to define pharmacological differences between NHPs and humans. For colorectal and cervicovaginal explants in both species, high concentrations of tenofovir (TFV) and maraviroc were predictive of anti-viral efficacy. However, their combinations resulted in increased inhibitory potency in NHP when compared to human explants. In NHPs, higher TFV concentrations were measured in colorectal versus cervicovaginal explants (p = 0.042). In humans, this relationship was inverted with lower levels in colorectal tissue (p = 0.027). TFV-resistance caused greater loss of viral fitness for HIV-1 than SIV. This, tissue explants provide an important bridge to refine and appropriately interpret NHP studies.

## Highlights

• Tenofovir-maraviroc combinations show greater potency in NHP than in human tissue
• Opposite drug distribution in mucosal tissues was observed between both species
• Greater loss of viral replication fitness with RT mutations for SIV than for HIV-1
• *Ex vivo* tissue models are a bridge between NHP studies and human clinical trials

## Graphical abstract

**Figure:**
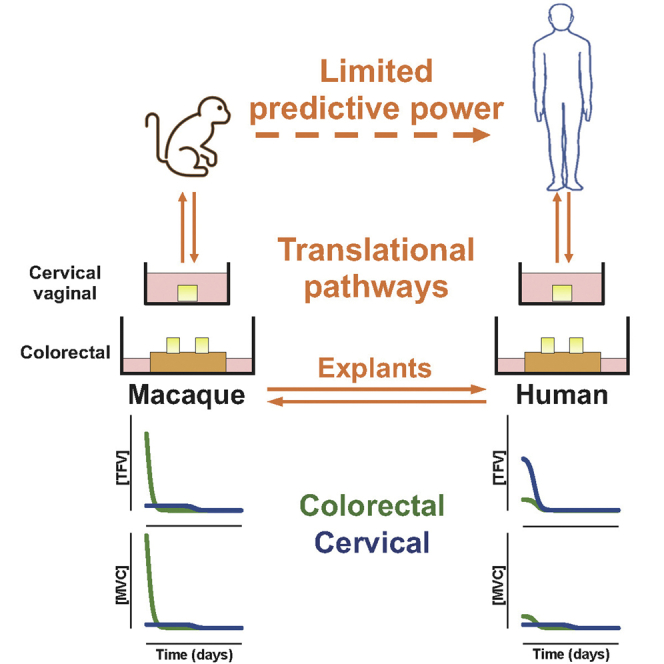

## Introduction

Antiretroviral (ARV)-based pre-exposure prophylaxis (PrEP) is an important strategy in reducing HIV-1 transmission rates and remains an important global public health priority. Despite the use of various animal models, including humanized mice (Hatziioannou and Evans, 2012) and sheep (Holt et al., 2015) for pre-clinical development, NHPs remain the most relevant challenge model to assess the potential efficacy of ARV prevention. However, dose-efficacy discrepancies between NHP studies and clinical trials, and between dosing routes, have been described (Romano et al., 2013; Anton et al., 2000). The gap in knowledge regarding the concentration-effect relationship in both species highlights the need to develop models that will facilitate comparison between NHPs and humans, thereby increasing the predictive capacity of NHP studies.

Many ARVs being considered for oral or topical PrEP, including the nucleotide reverse transcriptase inhibitor (NRTI) tenofovir (TFV), and the entry inhibitor (EI) maraviroc (MVC), are already used in highly active ARV treatment (HAART). For these drugs, a substantial amount of pharmacokinetic (PK) and pharmacodynamic (PD) data is available, including concentrations in blood plasma and genital secretions (Dickinson et al., 2010; Cohen et al., 2007). However, drug concentration measurements in blood plasma are not representative of mucosal tissue concentrations (Lederman et al., 2004; Trezza and Kashuba, 2014; Cohen et al., 2007; Brown et al., 2011; Dumond et al., 2007, 2009), and mucosal tissues are histologically and immunologically different from blood (Anton et al., 2000), affecting the expected correlation between concentration and efficacy at mucosal sites. In addition, drug accumulation is specific to each mucosal compartment, with differences between the intestinal and the female genital tract (Cohen et al., 2007; Louissaint et al., 2013; Patterson et al., 2011).

Assessment of concentration-efficacy correlations in mucosal tissues between species could help clarify discrepancies between human and NHPs data. However, this would require a significant number of NHPs for each candidate ARV and increase the complexity and size of clinical trials. Mucosal tissue explant models are an important tool for pre-clinical screening of PrEP regimens (Herrera and Shattock, 2014) and are increasingly being used in early clinical trials (McGowan et al., 2015, Fox et al., 2016; Anton et al., 2012; Richardson-Harman et al., 2012, 2014). Through tissue-associated drug pharmacological measurement and *ex vivo* challenge, the present study sought to assess the potential use of *ex vivo* mucosal tissue explants as a bridging model between NHPs studies and human clinical trials of anti-HIV PrEP candidates. Parallel studies with TFV and MVC were performed with NHP and human mucosal tissue explants to establish comparisons in the PK/PD relationship between both species.

## Results

### *In vivo* viral replication fitness is recapitulated in tissue explants

**Figure 1:**
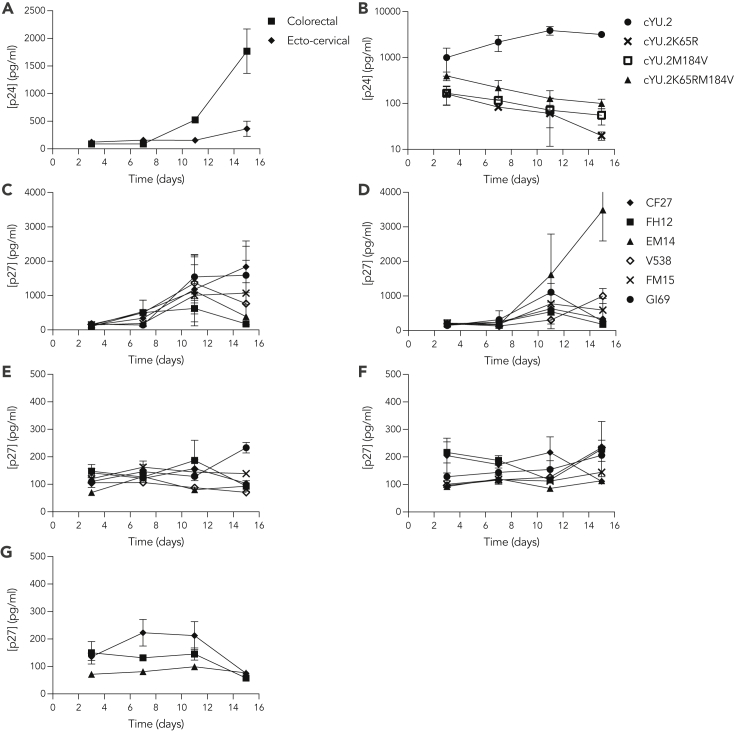
Replication fitness of HIV-1, SIV, and SHIV in human and NHP mucosal tissue explants Human (A) colorectal and ecto-cervical tissue explants were incubated with HIV-1_BaL_, or (B) colorectal explants were challenged with HIV-1_cYU.2_, HIV-1_cYU.2K65R_, HIV-1_cYU.2M184V_, HIV-1_cYU.2K65RM184V_. Rhesus macaque (C and D) colorectal, (E and F) vaginal, and (G) ecto-cervical explants were challenged with SIV_mac32H_ (C, E, and G) or RT-SHIV (D and F). After 2 h of challenge, explants were washed in PBS and cultured for 15 days. Supernatants were harvested at different time points and p24 or p27 concentrations measured by ELISA. Data are means ± SEM from n = 3 independent experiments performed with human explants in triplicate, and from n = 6 experiments with NHP colorectal and vaginal explants and n = 3 experiments with NHP ecto-cervical tissue, performed in duplicate.

We first established the viral replication fitness of subtype B R5-tropic HIV-1 isolates SIV_mac32H_ and RT-SHIV clones in human and Rhesus macaque mucosal tissue explants, respectively. All isolates infected colorectal and cervicovaginal explants (Figure 1) and, in both species, higher levels of viral replication were observed in colorectal explants compared with ecto-cervical and vaginal tissues. However, in some NHPs the peak of infection was observed at day 11 with a subsequent decrease in p27 levels.

ARV resistance is increasingly prevalent (Pennings, 2013; Snedecor et al., 2014) and can be associated with a decrease in viral replication capacity as observed for NRTI-resistant isolates. Hence, we generated a panel of NRTI-resistant clonal HIV-1_YU.2_ and SIV_mac32H_ isolates containing single point mutations in RT, K65R, and/or M184V (Figure S1) which have been well characterized in patients (Margot et al., 2006; White et al., 2002). When the study was conducted, L313T/I321V and V314T/I321V mutations were described as inducing resistance to MVC (Westby et al., 2007); however, when the mutations were introduced in HIV-1_YU.2_, no reduction of inhibitory potency was observed for MVC (Figure S1). We did not further evaluate these variants in the study. Mimicking the fitness loss observed *in vivo*, the three NRTI-resistant isolates showed reduced viral replication capacity in human explants compared to the wild-type clone (Figure 1B). However, in explants from some NHPs higher levels of viral replication were observed with SIV_mac32H M184V_ (Figure S2). Hence, the loss of replication capacity was not consistent when the same mutations were introduced in an SIV backbone and tested in NHP explants compared to an HIV-1 mutant used in human explants.

### Antiretroviral combinations are more potent in non-human primates than in human mucosal tissues

We evaluated the potency of TFV and MVC in mucosal tissue explants against challenge with the panel of HIV-1/SIV/SHIV isolates. TFV and MVC were applied topically, formulated alone or in combination in an aqueous hydroxyethylcellulose (HEC) gel; HEC gels are widely used for vaginal and rectal administration of drugs (Ciolacu et al., 2020). ARVs were applied 1 h before the challenge and removed 2 h post-challenge by washing in PBS. In NHP and human explants, greater potency was observed for TFV and MVC in colorectal tissue when compared to female genital tract tissue (Table 1) with median (IQR) fold decreased IC_50_ values for matched isolates of 1.8 (1.7–2.1). Although increased inhibition was observed for all treatments when TFV and MVC were dosed in combination vs individually, synergy was not observed (median Ψ estimates from non-competitive joint inhibition interaction model ranged from 1.1 to 3.6; Table 1). Interestingly, higher Ψ values of 3.6 and 2.7 (possibly indicative of antagonism) were observed in both human and NHP cervical explants, respectively. As expected, the potency of TFV was reduced against the mutant isolates, with no impact on the potency of MVC Table 1).

**Table 1:** ARV potency in explants across range of viral isolates

			IC_50_ (μM)^a^		Interaction
Specie	Tissue	Isolate	TFV	MVC	Ψ
Human	Cervical	HIV-1_BaL_	41.35 ± 0.57	2.85 ± 0.49	3.6
	Colorectal	HIV-1_BaL_	22.15 ± 1.39	1.26 ± 0.49	1.1
		HIV-1_YU.2_	28.86 ± 11.82	1.15 ± 0.84	N.R.
		HIV-1_YU.2 K65R_	62.37 ± 15.80	2.77 ± 1.12	N.R.
		HIV-1_YU.2 M184V_	37.85 ± 12.49	1.24 ± 1.88	N.R.
		HIV-1_YU.2 K65RM184V_	36.39 ± 3.83	1.43 ± 0.98	N.R.
NHP	Cervical	SIV_mac32H_	52.65 ± 41.49	2.74 ± 1.18	2.7
		SIV_mac32H K65RM184V_	81.30 ± 50.81	4.57 ± 2.92	N.R.
	Vaginal	SIV_mac32H_	56.14 ± 62.94	3.32 ± 3.19	1.2
		SIV_mac32H K65RM184V_	77.14 ± 31.54	3.31 ± 1.68	1.4
		RT-SHIV	52.15 ± 27.32	2.56 ± 1.59	1.5
	Colorectal	SIV_mac32H_	25.41 ± 5.35	1.96 ± 1.30	1.4
		SIV_mac32H K65RM184V_	47.89 ± 24.89	1.98 ± 1.07	1.8
		RT-SHIV	29.96 ± 13.25	2.32 ± 0.78	1.7
		SIV_mac32H K65R_	46.55 ± 12.60	2.12 ± 0.89	1.6
		SIV_mac32H M184V_	40.20 ± 30.10	3.37 ± 1.21	1.4

a Data are means (±SD) derived from three independent experiments performed in triplicate with human tissue and from independent experiments performed in duplicate with six macaques for wild-type isolates and at least three animals for resistant isolates. Median psi (Ψ) parameter where <1 indicates synergy, one additivity, and >1 antagonism.

### Pharmacology of tenofovir and maraviroc in non-human primates and human mucosal explants

**Figure 2:**
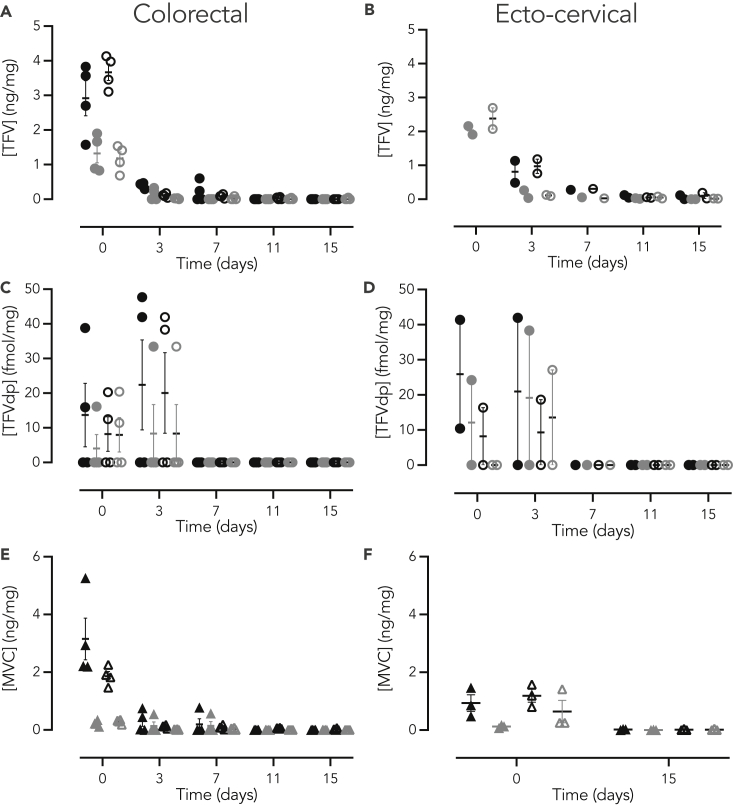
Concentrations of TFV and MVC in human mucosal tissue explants Human (A, C, and E) colorectal and (B, D, and F) ecto-cervical tissue explants were incubated for 3 h with TFV at 70 μM (●) or 7 μM (), MVC at 3.8 μM (▲) or 0.38 μM (), TFV 70 μM – MVC 3.8 μM (**○** and **▵**, respectively) or TFV 7 μM – MVC 0.38 μM ( and , respectively). Explants were harvested at different time points and concentrations of TFV, TFVdp or MVC measured. Data are median (IQR) derived from three independent experiments performed in duplicate.

**Figure 3:**
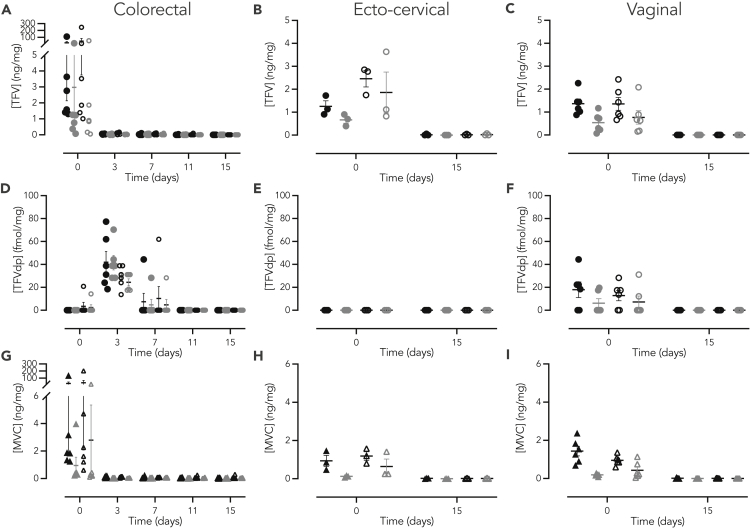
Concentrations of TFV and MVC in NHP mucosal tissue explants Rhesus macaque (A, D, and G) colorectal, (B, E, and H) cervical, and (C, F, and I) vaginal tissue explants were incubated for 3 h with TFV at 70 μM (●) or 7 μM (), MVC at 3.8 μM (▲) or 0.38 μM (), TFV 70 μM – MVC 3.8 μM (**○** and **▵**, respectively) or TFV 7 μM – MVC 0.38 μM ( and , respectively). Explants were harvested at different time points and concentrations of TFV, TFVdp or MVC measured. Data are median (IQR) derived from independent experiments performed with six macaques in duplicate.

Tissue concentrations of TFV and MVC and intracellular concentrations of the diphosphorylated active form of TFV (TFVdp) were measured following the 3 h *ex vivo* ARV exposure and viral challenge of explants. In human explants, average TFV exposure (AUC_3h-15d_) was significantly higher in ecto-cervical when compared to colorectal explants (fold change = 3.7, p = 0.002; Figure 2, Table S1). By comparison, MVC exposure was similar between tissue types (fold change = 1.3, p = 0.655). In NHP explants, exposure for both ARVs trended higher in colorectal explants than in ecto-cervical (fold change = 2.5, p = 0.470 for TFV and fold change = 3.8, p = 0.201 for MVC) and vaginal tissues (fold change = 4.4, p = 0.373 for TFV and fold change = 4.3, p = 0.209 for MVC) without reaching statistical significance (Figure 3, Table S2). No significant differences were observed for these PK parameters between ecto-cervical and vaginal explants. Notably, when comparing the ARV concentrations measured in human and macaque tissues, *C*_*max*_ for TFV and MVC were significantly higher in human ecto-cervical explants than in macaque tissue (fold increase: 5.6, p = 0.003 for TFV and 2.2, p = 0.032 for MVC), and there was a trend to lower *C*_*max*_ values for both drugs in human colorectal explants than in macaque tissue (fold increase: 8.4, p = 0.221 for TFV and 11.5, p = 0.240 for MVC). With the harvesting schedule of the assay, TFVdp was neither detected in humans nor in NHP ecto-cervical explants. However, this analyte was found in NHP vaginal tissue explants at t_0_, and the C_max_ was lower than in colorectal explants (fold increase: 1.6, p = 0.020). No significant differences in TFVdp concentrations were found in colorectal explants between NHP and humans.

When comparing the different *ex vivo* dosing concentrations, significantly higher PK parameters were measured in both human tissues after exposure to high concentration gels with TFV (p < 0.0001) (AUC_3h-15d_ fold increase: 6.6 in ecto-cervical explants, 2.9 in colorectal tissue) and with MVC (AUC_3h-15d_ fold increase: 4.3 (p = 0.0002) in ecto-cervical explants; 5.4 (p < 0.0001) in colorectal tissue). In macaque explants, this high correlation between ARV concentration exposure-high tissue drug concentration was also observed; however, statistical significance was only reached for both drugs in vaginal explants (AUC_3h-15d_ 2.1-fold increase for TFV, p = 0.006; 3.8-fold increase for MVC, p < 0.0001), and for MVC in ecto-cervical explants (AUC_3h-15d_ 14.1-fold increase, p < 0.033). In human colorectal explants, high TFV PK values tended to correlate with greater concentrations of TFVdp. This correlation was neither observed in NHP colorectal nor in vaginal explants.

No effect was observed on PK parameters of each drug when human explants were treated with the ARV-combination. However, in NHPs explants, combining the two ARVs resulted in an increase of these parameters for both drugs reaching statistical significance only for TFV in ecto-cervical explants (AUC_3h-15d_ 2.2-fold increase, p = 0.036) (Figure 3, Table S2).

The p24 and p27 concentrations at different time points during the 15 days of culture allowed us to calculate p24 or p27 AUCτ between days 3 and 15 of culture (p24/p27 AUC_3-15_) (Table S3). In general, and as expected following explant dosing with ARVs, lower p24/p27 AUC_3-15_ values were measured in ARV-dosed explant cultures than in untreated samples. On average, a further decrease was observed with combinations in comparison to each individual drug. For each ARV, tested alone and in combination, the lowest p24/p27 AUC_3-15_ were obtained with the higher concentration gels as greater levels of inhibition were reached. Specifically, the decrease of p24 AUC_3-15_ values between the drugs used in combination and tested alone reached statistical significance for TFV against HIV-1_YU.2_ (p = 0.049 at high concentration) and resistant HIV-1_YU.2_ isolates (p = 0.046 at high concentration against HIV-1_YU.2 K65R_, p = 0.012 at high concentration against HIV-1_YU.2 M184V_, p = 0.020 at low concentration against HIV-1_YU.2 K65RM184V_) in colorectal tissue. Further reductions were measured for the two ARVs alone and in combination with the high compared to the low concentration gel with statistical significance in colorectal explants against HIV-1_YU.2_ (p = 0.005 for TFV-MVC), HIV-1_YU.2 K65R_ (p = 0.001 for TFV, p = 0.003 for MVC, p = 0.001 for TFV-MVC), HIV-1_YU.2 M184V_ (p = 0.0006 for TFV, p = 0.010 for MVC, p = 0.007 for TFV-MVC), and HIV-1_YU.2 K65RM184V_ (p = 0.0003 for TFV, p = 0.026 for MVC, p = 0.010 for TFV-MVC). The decrease of p27 AUC_3-15_ values with the ARV-combination reached statistical significance for MVC in colorectal explants against SIV_mac32H_ (p = 0.005 at high concentration, and p = 0.003 at low concentration). When greater anti-viral activity was obtained with an increase of the dosing concentration, lower p27 AUC_3-15_ values were also calculated with significant differences for the two drugs alone and/or in combination in colorectal explants against SIV_mac32H_ (p = 0.0005 for TFV, p < 0.0001 for MVC, p = 0.0005 for TFV-MVC), RT-SHIV (p = 0.032 for TFV, p = 0.042 for TFV-MVC) and SIV_mac32H K65RM184V_ (p = 0.049 for TFV-MVC).

For both drugs, the reduction in the p24/p27 AUC_3-15_ values was greater in colorectal tissue than in ecto-cervical explants in parallel with the IC_50_ at day 15 (Table 1), demonstrating that this difference between both mucosal tissues was consistent during the 15 days of culture.

### *Ex vivo* drug concentration and infectivity inversely correlate in explants

**Figure 4:**
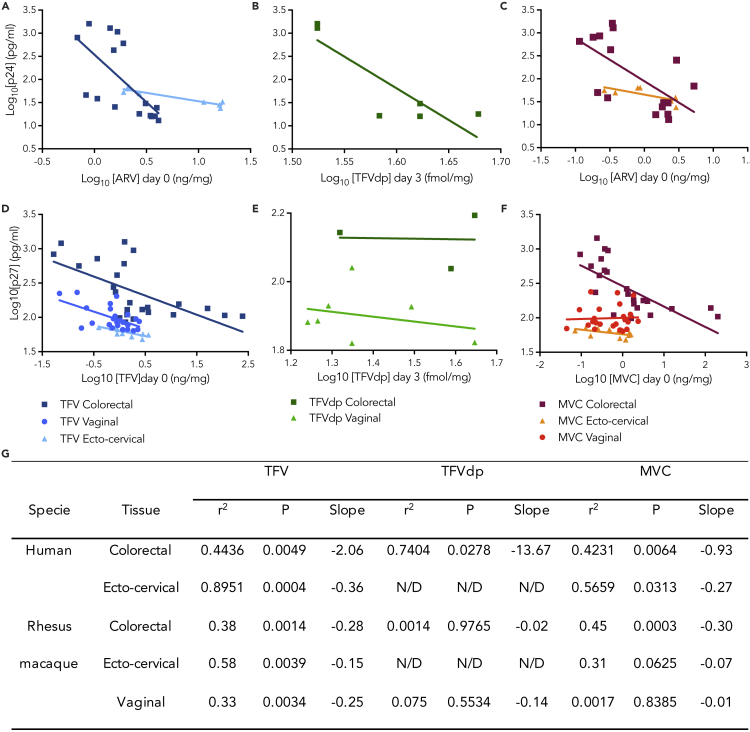
*Ex vivo* drug concentration and infectivity inversely correlate in explants Linear correlations in solid lines are shown for C_*max*_ values of (A and D) TFV at day 0, (B and E) TFVdp found at day 3 and (C and F) MVC at day 0 correlated with p24 (A, B, and C) and p27 (D, E, and F) concentrations measured at day 15, after *ex vivo* exposure of tissue explants to gels containing high or low concentrations of drug alone or in combination; and challenge with HIV-1_BaL_. (G) Correlations were assessed using a Pearson correlation test. N/D: not detectable for tissues where all observations were below the limits of quantification (BLQ). The data are derived from at least three independent experiments (n = 3 for human tissues, n = 6 for NHP colorectal and vaginal explants and n = 3 for NHP ecto-cervical tissue) performed in duplicate.

Negative correlations between explant drug concentration and HIV p24 or SIV p27 concentrations were found in human and NHP explants (Figure 4), demonstrating the relationship between increased tissue drug concentrations and lower levels of infection in the *ex vivo* model. Notably, however, in human explants, we observed greater negative values for the slope of the linear correlation than in NHPs (Figure 4G).

The PK-PD linear correlation for TFV was statistically significant in all human and NHP tissue types (Figure 4G). Hence, we decided to further investigate these correlations by assessing if a non-linear correlation would be a better fit. Analysis revealed that a non-linear fit was possible (Table S4). However, statistical significance was only reached in ecto-cervical human explants and in vaginal NHP explants.

## Discussion

Here, we have demonstrated discrepancies in ARV exposure and potency between species (human vs NHP) and tissue type (female genital vs colorectal) in the mucosal tissue explant model. The order of potency in tissue explants with TFV and MVC was the same as that described in the literature in cellular models (Herrera et al., 2016) and *in vivo* in NHPs (Dobard et al., 2015), and it was maintained in all tissue models and in both species (Table 1). Furthermore, and as reported previously in NHPs (Dobard et al., 2015), when the drugs were tested in combination in the explant model there was an increase in inhibitory activity for both drugs in NHPs and humans. However, increased inhibition tended to be higher in NHP vs human explants and lower in cervical vs vaginal and colorectal explants and did not meet our definition of synergy within any explant condition. These differences could be related to lower level of viral replication in cervicovaginal tissue explants (Figures 1A and 2), which mimics the lower *in vivo* susceptibility of female genital tract to HIV infection compared to colorectum. The relatively high vulnerability of the colorectal tract to HIV-1 transmission is likely owing to histological and immunological differences between intestinal and genital mucosae. Colorectal mucosa has a single-cell columnar epithelium in contrast to the pluri-stratified squamous epithelium of the lower female genital tract. Moreover, intestinal lamina propia contains an abundance of highly activated target cells for HIV infection, can transfer infectious virus to the underlying lymphoid tissue and is the major site of viral replication and CD4 T cell depletion during acute infection (Anton et al., 2000; Lapenta et al., 1999; Poles et al., 2001). The loss of viral fitness described *in vivo* for NRTI-resistant isolates (McColl et al., 2004; Lloyd et al., 2016) was also mimicked in tissue explants (Figures 1B and 2). However, this model revealed differences in the viral replication capacity with a more pronounced loss for HIV-1 mutants tested in human explants than for resistant SIV isolates in NHP explants. The viral replication differences observed between humans and NHP could be linked to immunological specificities. Different immune cell type frequencies have been described for both species in blood (Bjornson-Hooper et al., 2019), which could affect the mucosal immune content. Another factor that has been described to affect HIV susceptibility and ARV PK/PD in mucosal compartments is the microbiota (Abdool Karim et al., 2019). Microbial communities have been described to differ between humans and NHPs (Chen et al., 2018). However, in our model, the sustained use of antibiotic and antifungal cocktail before and after dosing and challenge annul this factor. The activity of MVC depends on its binding to CCR5; despite differences in the kinetics of dissociation described between rhesus and human CCR5 (Napier et al., 2005), NHP studies (Massud et al., 2013) and clinical trials (Coll et al., 2015; Fox et al., 2016; McGowan et al., 2019; McGowan et al., 2022; Sekabira et al., 2021) have shown limited protective potency even in the presence of MVC at levels above the minimum effective concentration. Pre-clinical (Herrera et al., 2016; Fletcher et al., 2016) and clinical (McGowan et al., 2019; Sekabira et al., 2021) studies using the *ex vivo* challenge model have shown that frequent repeat dosing or combination with other ARVs increases the inhibitory activity of MVC.

We assessed the combinatorial activity (synergy/additivity/antagonism) of TFV and MVC using a previously published non-competitive joint inhibition model (Chakraborty and Jusko, 2002). Although we saw increased inhibitory activity by our combination treatment, we did not observe a synergistic interaction. Given that previous studies with cell culture models have demonstrated synergy between TFV and MVC (Srinivas et al., 2020), this finding may be specific to explant systems and could be attributable to early physiologic changes that have been described in cultured tissues that can influence ARV potencies such as decreased CCR5 expression and deoxynucleotide concentrations (Nicol et al., 2015).

Differences in TFV and MVC exposure were also observed in our *ex vivo* tissue explant model. For both ARVs, exposure (i.e., AUC_3h-15d_) was higher in cervicovaginal and colorectal NHP explants when compared to human tissue (Tables S1 and S2). Importantly, lower TFV exposure was observed in NHP ecto-cervical than in colorectal explants and the opposite distribution was measured in human explants. Higher intracellular TFVdp concentrations have been found in rectal tissue than in vaginal tissue of pigtail macaque (Radzio et al., 2012) similar to our results with mucosal explants from rhesus macaque. These results further correlate with an NHP study where the drug concentration measured in vaginal fluid after vaginal dosing was lower than in rectal fluid following rectal dosing (Nuttall et al., 2012). Thus, *in vivo* NHP studies might overestimate the amount of drug required to obtain equivalent colorectal dosage in humans and underestimate the amount required for ecto-cervical tissue. These pharmacological differences are linked to multiple factors, in addition to microbiome, such as drug transporters, whose expression patterns are not only tissue-dependent but also distinct between NHPs and humans. Lower levels of ARV efflux transporters have been described in the NHP female genital tract compared to humans (Hijazi et al., 2020) which explain the distinct ARV retention levels measured in our study. Furthermore, and in parallel to our results, specie-dependent drug distribution has also been observed in the gut following oral dosing of macaques and humans with several ARVs including TFV and MVC among others (Akabane et al., 2010; Thompson et al., 2019). Another important factor in the pharmacology of TFV is the activity of the kinases involved in the intracellular phosphorylation of TFV resulting in the active diphosphorylated metabolite, TFVdp. This phosphorylation occurs in two steps, adenylate kinase 2 (AK2) phosphorylates TFV to TFV-monophosphorylated (mp) in female genital tract and colorectal tissue; while phosphorylation of TFVmp to TFVdp in colorectal tissue is catalyzed by creatine kinase, muscle (CKM), and by pyruvate kinase muscle (PKM) and pyruvate kinase liver and red blood cell (PKLR) in vaginal tissue (Lade et al., 2015). Furthermore, AK2 has a 100% between humans and rhesus macaques (https://www.uniprot.org/); however, to our knowledge, no study has assessed if the 90% homology between these two species for the other three kinases affects the efficacy of TFV metabolization. These factors could impact the predictive power of animal models and should be further studied in comparative tissue explant studies.

However, the lack of significant differences in PK parameters for TFV and MVC between ecto-cervical and vaginal explants from NHPs (Table S2) is in accordance with a study where after topical vaginal application of TFV in women no differences were observed in drug concentrations between proximal and distal areas of the female genital tract (Schwartz et al., 2011). A recent publication by Nicol et al. showed that peak expression of the intracellular active metabolite TFVdp is detected between 24 and 48 h after TFV dosing (Nicol et al., 2015). Our harvest schedule on days 0, 3, 7, 11, and 15 of explant culture was defined before that publication, resulting in the measurement of tissue TFVdp during its decay phase. Nevertheless, this schedule exhibited greater persistence of metabolite in colorectal explants, where the metabolite was still detected up to 3 days post-dose vs just 3 h post-dose in vaginal tissue. This could be owing to the differential expression of kinases and nucleosidases responsible for adding and removing phosphate groups on nucleotide analogs (Hu et al., 2014). The explant model also recapitulated the higher concentrations of TFVdp in colorectal tissue compared to cervicovaginal mucosa measured in various clinical trials (Karim et al., 2011).

Macaques remain the main challenge model for HIV/AIDS; however, pre-clinical data for the prioritization of ARVs and vaccines need to be supplemented with the evaluation of other factors than protection against *in vivo* challenge of macaques, to predict efficacy in clinical trials. Refinement of NHP models and further characterization in tissue explant models of key factors affecting the pharmacology of HIV prevention strategies are needed. Using only human tissue explants to prioritize candidate ARVs would involve financial, time, and ethical constraints linked to the recruitment of a large number of participants. *Ex vivo* challenge of tissue explants would allow conversion between the tissue drug concentrations needed to obtain protection in NHPs with the concentration required for efficacy in human tissue. Hence, the tissue explant model may provide an important bridge between NHP PrEP studies and human clinical trials, refining the NHP model to enhance the predictive utility of NHPs and reducing the risk of late-stage failure in clinical trials.

### Limitations of the study

The unavailability of human vaginal tissue in this study represents a limitation to establish the potential effect of the intrinsic variability within the human cervicovaginal compartment.

The main limitation of this study is the relatively small sample size, with no opportunity to evaluate other drug concentrations, ARVs, and viral isolates in specimens such as ecto-cervical tissue. The choice of RT-SHIV, instead of an Env-SHIV, was taken on the basis that results obtained with RT-SHIV could potentially serve for the design of combination-based prevention strategies including TFV or MVC and non-NRTI ARVs, which are not active against all SIV isolates (Ambrose et al., 2004; Isaka et al., 2000). Although the results obtained in this study might not be representative of the broad range of ARVs currently in the HIV prevention pipeline, the evaluation of two ARVs with different mechanisms of action and cellular transport/efflux give sufficient evidence to highlight the need to adapt the pre-clinical criteria of the selection of candidate ARVs to each drug and to its activity in different mucosal tissues from humans or NHPs.

The explant model has limitations, including (i) progressive loss of architecture despite the maintenance of CD4:CD8 T cell ratios and sufficient viability to sustain viral replication for more than 10 days (Fletcher et al., 2006); (ii) paucity of data regarding the preservation of immune competence (Grivel and Margolis, 2009); (iii) limitation to demonstrate sterilizing protection; and (iv) inability to metabolize certain prodrugs such as tenofovir disoproxil fumarate which is the formulated version of TFV for oral administration.

## STAR★Methods

### Key resources table

**Table:** 

REAGENT or RESOURCE	SOURCE	IDENTIFIER
**Bacterial and virus strains**		

HIV-1 BaL	NIH AIDS reagent program	ARP-510
RT-SHIV	Soderberg, K. et al., 2002	N/A

**Biological samples**		

Human colorectal fresh tissue	Imperial College Healthcare Tissue Bank	https://www.imperial.ac.uk/imperial-college-healthcare-tissue-bank/
Human ecto-cervical fresh tissue	Imperial College Healthcare Tissue Bank	https://www.imperial.ac.uk/imperial-college-healthcare-tissue-bank/
Human peripheral blood mononuclear cells	NHS Blood and Transplant	NC24 Leucocyte cone

**Chemicals, peptides, and recombinant proteins**		

Hydroxyethylcellulose (HEC) (Natrosol^TM^ 250 HX pharm)	Ashland / Aqualon	N/A
Maraviroc free base (UK-427857, PF-3419979)	Pfizer Ltd	Lot 0008
Maraviroc	NIH AIDS reagent program	ARP-11580
Tenofovir (PMPA, TFV, GS-1278)	CONRAD	N/A
Tenofovir	NIH AIDS reagent program	ARP-10199
Emtricitabine (FTC)	NIH AIDS reagent program	ARP-10071

**Critical commercial assays**		

HIV-1 p24 ELISA	AIDS Vaccine Program, National Cancer Institute	N/A
HIV-1 p24 Antigen ELISA, RETROtek, Zeptometrix	Gentaur	0801200
SIV p27 Antigen ELISA, RETROtek, Zeptometrix	Gentaur	0801201
QuickChange Lightning Site-Directed Mutagenesis Kit	Agilent	210514
Lipofectamine 2000	Life Technologies	11668019
Luciferase assay system	Promega	E4530
Precellys® hard tissue reinforced metal beads kit (MK28-R)	Cayman Chemical	16858

**Experimental models: Cell lines**		

TZM-bl cells	NIH AIDS reagent program	ARP-8129
HEK 293T cells	ATCC	CRL-3216
C8166 cells	NIBSC-CFAR	ARP013

**Experimental models: Organisms/strains**		

Rhesus macaque (*Macaca mulatta*)	Tulane National Primate Research Center	N/A

**Oligonucleotides**		

Primer pYU.2-K65R-up: 5′-CTCCAGTATTTGCCATA AAGAGAAAAGACAGTACTAAATGGAG-3′	This paper	N/A
Primer pYU.2-K65R-down: 5′-CTCCATTTAGTACTGT CTTTTCTCTTTATGGCAAATACTGGAG-3′	This paper	N/A
Primer pYU.2-M184V-up: 5′-CCAGACCTAGTTATCT ATCAGTACGTAGATGATTTGTACGTAGG-3′	This paper	N/A
Primer pYU.2-M184V-down: 5′-CCTACGTACAAAT CATCTACGTACTGATAGATAACTAGGTCTGG-3′	This paper	N/A
Primer pJ5-K65R-up: 5′-CACCCCCACATTTGCTAT AAAGAGAAAAGATAAGAACAAATGGAG-3′	This paper	N/A
Primer pJ5-K65R-down: 5′-CTCCATTTGTTCTTATC TTTTCTCTTTATAGCAAATGTGGGGGTG-3′	This paper	N/A
Primer pJ5-M184V-up: 5′-GATGTGACCTTAGTCC AGTATGTAGATGACATCTTAATAGCTAGTG-3′	This paper	N/A
Primer pJ5-M184V-down: 5′-CACTAGCTATTAAGA TGTCATCTACATACTGGACTAAGGTCACATC-3′	This paper	N/A

**Recombinant DNA**		

pHIV-1 YU2	NIBSC-CFAR	100 840
pHIV-1 YU2 K65R	This paper	N/A
pHIV-1 YU2 M184V	This paper	N/A
pHIV-1 YU2 K65R M184V	This paper	N/A
pJ5 del T-KS-(SIVmac32H)	NIBSC-CFAR	ARP229
pSIVmac32H K65R	This paper	N/A
pSIVmac32H M184V	This paper	N/A
pSIVmac32H K65R M184V	This paper	N/A

**Software and algorithms**		

GraphPad Prism (v.8)	GraphPad Software, Inc.	https://www.graphpad.com/
SAS v9.5	SAS Institute Inc.	https://www.sas.com
R (v. 3.6.1)	R Code Team and R Foundation for Statistical Analysis	https://www.r-project.org/
AB Sciex Analyst Chromatography Software (v. 1.6.2)	Sciex	https://sciex.com

### Resource availability

#### Lead contact

Further information and requests for resources and reagents should be directed to and will be fulfilled by the lead contact, Carolina Herrera (cherrer1@imperial.ac.uk).

#### Materials availability

Plasmids generated in this study can be access upon request to the lead contact.

### Method details

#### Animal welfare

Six female Rhesus macaques (*Macaca mulatta*) of different ages (CF27: 13 years, FH12: 8 years, EM14: 9 years, V538: 16 years, FM15: 11 years, GI69: 6 years) were included in the study. Animals were not treated with Depo-Provera. Macaques were humanely euthanized with ketamine hydrochloride (10 mg/kg) and tiletimine / zolazepan (Telazol, 8 mg/kg) in accordance with the American Veterinary Medical Association Guidelines on Euthanasia, 2013.

#### Human tissues

Cervical tissue was obtained from adult pre-menopausal patients undergoing planned therapeutic hysterectomies with nonmalignant pathology or posterior and anterior vaginal repair surgery at St. Mary’s Hospital, Imperial College of London, United Kingdom. Colorectal tissue was obtained from patients undergoing rectocele repair and colectomy from colorectal cancer at St. Mary’s Hospital (London, United Kingdom). Only healthy tissue obtained at 10 to 15 cm away from any tumor was employed.

#### Reagents and plasmids

Aqueous HEC gels containing TFV (0.1 and 1% w/w), MVC (0.01 and 0.1% w/w), and two TFV + MVC combination gels (0.1 + 0.01% w/w, and 1 + 0.1% w/w) were manufactured as described previously (Malcolm et al., 2013).

HIV-1_BaL_ (Gartner et al., 1986) was provided by the NIH AIDS Research & Reference Reagent Program (http://www.aidsreagent.org/). RT-SHIV (Soderberg et al., 2002) was kindly donated by Dr. Stahl-Hennig (DPZ, Germany). Full-length, replication and infection-competent proviral HIV-1_YU.2_ clone, pYU.2 (Li et al., 1992), and proviral SIV_mac32H_, pJ5 (Rud et al., 1994) were provided by the Center for AIDS Reagents (http://www.nibsc.org/). Infectious HIV-1 and SIV clones with K65R and/orM184V mutations were constructed as previously described (Garcia-Perez et al., 2008). Briefly, site-directed mutations were introduced by PCR in pYU-2 and pJ5 clones using the QuickChange Lightning Site-Directed Mutagenesis Kit (Agilent Technologies, Santa Clara, CA) and the mutagenic primers following manufacturer instructions. Clones were sequenced to verify the mutations had been inserted.

#### Cell, tissue explants and virus culture conditions

All cell and tissue explant cultures were maintained at 37°C in an atmosphere containing 5% CO_2_. TZM-bl cells (Wei et al., 2002; Derdeyn et al., 2000; Platt et al., 1998) and human epithelial kidney (HEK) 293T cells (Pear et al., 1993, DuBridge et al., 1987) were grown in Dulbecco’s Minimal Essential Medium (DMEM) (Sigma-Aldrich, Inc., St. Louis, MO) and C8166 cells in RPMI 1640 medium (Sigma-Aldrich), both containing 10% fetal calf serum (FCS), 2mM L-glutamine and antibiotics (100 U of penicillin/ml, 100 μg of streptomycin /ml, and for HEK 293T cells, 0.5 mg/ml of the neomycin analog G-418). Both cell lines were tested for mycoplasma contamination and confirmed mycoplasma-free.

Mucosal tissue specimens were transported to the laboratory and processed less than 1 h after resection. Upon arrival in the laboratory, resected tissue was cut into 2–3 mm^3^ explants comprising epithelial and stromal layers for ecto-cervical and vaginal tissue, or epithelium and muscularis mucosae for colorectal tissue, as described previously (Herrera et al., 2009; Hu et al., 2004). Tissue explants were maintained with DMEM containing 10% fetal calf serum, 2 mM L-glutamine and antibiotics (100 U of penicillin/ml, 100 μg of streptomycin /ml, 80 μg of gentamicin /ml).

Recombinant vectors and SIV_mac32H_ were transfected in HEK293T cells with Lipofectamine 2000 following manufacturer’s instructions (Life Technologies, Carlsbad, CA). The laboratory-adapted isolate HIV-1_BaL_ was passaged for 11 days through PBMCs activated as described previously (Gordon et al., 1999).

#### Infectivity and inhibition assays in tissue explants

The infectivity of virus preparations was estimated in TZM-bl cells (by luciferase quantitation of cell lysates, Promega, Madison, WI) and PBMCs for HIV-1 isolates or C8166 cells for SIV clones (by measurement of p24 or p27 antigen content in cell culture supernatant, respectively). The extent of luciferase expression was recorded in relative light units (r.l.u) as described previously (Herrera et al., 2009). Viral p24 content in supernatant was measured with HIV-1 p24 ELISA (Zeptometrix Corporation, Buffalo, NY) and p27 with SIV p27 ELISA (Zeptometrix Corporation) following manufacturer’s instructions. Viral growth was reported as pg/ml of p24/p27, extrapolated from the p24/p27 kit-supplied standard curve generated by ODs using a sigmoidal dose-response (Prism, GraphPad). Appropriate dilutions of culture supernatants were applied to ensure data was within the 95% interval of the standard OD range.

Inhibition assays were performed using a standardized amount of virus culture supernatant normalized for infectivity. HEC gels containing TFV, MVC or the combination TFV-MVC were prepared and used at a concentration above the IC_50_ (TFV 70 μM and/or MVC 3.8 μM) and at one below the IC_50_ (TFV 7 μM and/or MVC 0.38 μM). Gels of high and low % were diluted in PBS to the desired high and low concentrations for each drug, respectively. Tissue explants were incubated with drug for 1 h before virus was added for 2 h at 10^4^ TCID_50_/ml. Explants were then washed four times with PBS to remove unbound virus and drug. Cervicovaginal explants were then transferred to fresh microtiter plates and colorectal explants were transferred onto gelfoam rafts (Welbeck Pharmaceuticals, UK). Tissue explants were cultured in the absence of drug for 15 days and maintained by harvesting approximately two-thirds of culture supernatant at days 3, 7, 11 and 15, and refeeding the cultures with fresh medium. The extent of virus replication in tissue explants was determined by measuring the p24 antigen concentration for HIV-1 and p27 for SIV and RT-SHIV, in supernatants at each harvest time point (HIV-1 p24 ELISA and SIV p27 ELISA, Zeptometrix Corporation). The lower limit of quantification (LLOQ) of the assay was 1.95 pg of p24 or 15.62 pg of p27/ml. The inhibitory potency of the drugs at each concentration was measured at day 15 and, having tested only two concentrations in this pilot study, we estimated the IC_50_ with a linear regression. The percentage of inhibition by the drugs was normalized relative to p24/p27 values obtained for explants not exposed to virus or compound (0% infectivity) and for explants infected with virus in the absence of drug (100% infectivity).

#### Drug concentrations in tissue explants

To measure drug concentrations during the 15 days of culture of the tissue explants, infectivity assays were set up in replicates to define a baseline of drug level (t_0_) (after 3 h of dosing/viral challenge and PBS wash to remove unbound drug and virus) and for each time point of culture supernatant harvest (days 3, 7, 11 and 15). Due to the limited size of the macaque ecto-cervical and vaginal specimens, sparse sampling was performed including explant harvesting at t_0_ and day 15. Extracellular tissue concentrations of TFV and MVC and intracellular for TFV-DP were measured (Veselinovic et al., 2014) and converted to ng/mg for TFV and MVC and to fmol/mg for TFV-DP.

Drug concentrations were measured in all matrices using LC-MS/MS methods with ± 15% [20% at the lower limit of quantification (LLOQ)] precision and accuracy. Frozen tissue biopsies were weighed then homogenized in Precellys® hard tissue grinding kit tubes (Cayman Chemical, MI, USA) with cold 70:30 acetonitrile / 1 mM ammonium phosphate buffer (pH 7.4). Following protein precipitation extraction with labeled internal standards (^13^C TFV, ^13^C TFVdp and maraviroc-d_6_). For quantification of TFV and TFVdp, TFV was eluted from a Waters Atlantis T3 (100 x 2.1 mm^2^, 3 μm particle size) analytical column, and TFVdp was eluted from a Thermo Biobasic AX (50 x 2.1 mm^2^, 5 μm particle size) analytical column. An API-5000 triple quadrupole mass spectrometer was used to detect all analytes. Data were collected using AB Sciex Analyst Chromatography Software. The dynamic range of this assay was 0.02–20 ng/mL of homogenate for TFV and TFVdp using a 1/concentration weighted linear regression. To convert volume to mass, tissue density was assumed to be 1.06 g/cm^3^. Concentrations were ultimately converted into ng/mg (TFV) or fmol/mg (TFVdp) tissue for final reporting. To measure MVC, the resulting protein extract was analyzed on a Shimadzu Prominence HPLC by reverse phase chromatography with a Phenomenex Synergi Polar-RP column (50x2mm, 2.5 um partical size). Detection of the analyte and internal standard used electrospray ionization in the positive mode on an AB Sciex API-5000 triple quadrupole mass spectrometer. The dynamic range of the MVC assay was 0.600–1500 ng/mL of tissue homogenate. All methods were validated as mandated by the industry guidance set by the US DHHS et al., 2001.

#### Pharmacological data analysis

PK and PD parameters were estimated using the tissue-associated drug levels at different time points of explant culture. Concentrations that were detectable but below the limit of quantification were imputed as 50% of the lower limit of quantification (LLOQ) for the analyte and matrix. Concentrations that were below the limit of detection were considered as “0”. Measurement of extracellular concentrations of TFV and MVC and of intracellular concentrations of the diphosphorylated active form of TFV (TFVdp), allowed calculation of PK parameters including, the area under the curve (AUC) between 3 h and day 15 (AUC_3h-15d_). and the peak or highest concentration (C_*max*_). For TFV and MVC, C_*max*_ was observed at t_0_. However, taking into account that TFVdp is a sub-product of TFV which is diphosphorylated in the cellular cytoplasm, TFVdp was not detectable at t_0_; instead, C_*max*_ was calculated at the second point of our harvesting schedule, day 3. The *C*_max_ was estimated directly from experimental data. The AUC_3h-15d_ after dose was estimated using the log-linear trapezoidal method (Prism, GraphPad). To quantify the pharmacodynamic interaction between TFV and MVC, a previously published non-competitive joint inhibition model (Chakraborty and Jusko, 2002) of the following form was fit to estimate the potency factor Ψ, which is an empirical interaction term, where Ψ = 1 demonstrates additive effects, Ψ < 1 is synergism and Ψ > 1 antagonism (Equation 2). C is the observed tissue concentration of TFV or MVC, E is the AUC_3h-15d_ of p24 or p27, and by fixing the 50% effective concentration (EC_50_) and Hill slope (H) parameters from Equation 1.

(Equation 1) $$E=E_{0}+\frac{C^{H}\times E_{max}}{C^{H}+EC_{50}^{H}}$$

(Equation 2) $$E\;=\frac{{\left(\frac{C_{TFV}}{\psi \;\times EC_{50,TFV}}\right)}^{H_{TFV}}+{\left(\frac{C_{MVC}}{\psi \;\times EC_{50,MVC}}\right)}^{H_{MVC}}+\;{\left(\frac{C_{TFV}}{\psi \;\times EC_{50,TFV}}\right)}^{H_{TFV}}\times \;{\left(\frac{C_{MVC}}{\psi \;\times EC_{50,MVC}}\right)}^{H_{MVC}}}{1+\;{\left(\frac{C_{TFV}}{\psi \;\times EC_{50,TFV}}\right)}^{H_{TFV}}+\;{\left(\frac{C_{MVC}}{\psi \;\times EC_{50,MVC}}\right)}^{H_{MVC}}+\;{\left(\frac{C_{TFV}}{\psi \;\times EC_{50,TFV}}\right)}^{H_{TFV}}\times \;{\left(\frac{C_{MVC}}{\psi \;\times EC_{50,MVC}}\right)}^{H_{MVC}}}$$

The p24 or p27 AUCτ between days 3 and 15 of culture (p24/p27 AUC_3-15_) were estimated with the non-cumulative viral antigen concentrations at the different time points of supernatant harvest between days 3 and 15 and using the log-linear trapezoidal method (Prism, GraphPad).

#### Statistics

Drug concentrations were log_10_ transformed and correlated with the corresponding log transformed p24 level at day 15 of tissue explant culture post-infection for each animal or subject using a Pearson correlation test. *P* values were determined using a two-tailed unpaired Student t test, and *P* < 0.05 was considered statistically significant. TFV measures from cervical, vaginal and colorectal tissues were log_10_ transformed and paired with the corresponding explant infectibility result (i.e. log_10_ p24 or p27) for each sample. Paired TFV and p24 or p27 endpoints were entered into a three parameter, log-log, Hill slope, non-linear model (Equation 3) where the fit of the model was tested by nonlinear least-squares ANOVA and the proportion of variance that each model explained (*r*^*2*^) was calculated [i.e. (1-) the sum of the squared distances from each fitted curved divided by the squared distances from a horizontal line].

(Equation 3) $${\text{Log}}_{10}\left(\text{p}24/\text{p}27\right)=\text{b}+\left(\text{a}-\text{b}\right)/1{+10}^{\left(\left(\text{Log}10\text{Dose}\right)-\text{c}\right)})$$

The fit of each three-parameter non-linear model was compared to an alternative four parameter model using the information criterion of Akaike (AIC), where a lower AIC indicates improved model fit (Glatting et al., 2007).

#### Study approval

All animal studies were approved by the Tulane National Primate Research Center (TNPRC) Institutional Animal Care and Use Committee (IACUC). TNPRC is accredited by the Association for Assessment and Accreditation of Laboratory Animal Care (AAALAC no. 000594). The TNPRC Office of Laboratory Animal Welfare (OLAW) assurance number is A4499-01 and U.S. Department of Agriculture registration number is 72-R-0002. All human tissues were collected after receiving signed informed consent from all patients and under protocols approved by the Local Research Ethics Committee.

## Data Availability

• All data reported in this paper will be shared by the lead contact upon request. • Any additional information required to reanalyze the data reported in this paper is available from the lead contact upon request. • No code was generated for this study.
