# Supplementary material for: The ex vivo pharmacology of HIV-1 antiretrovirals differs between macaques and humans
Source: iScience. 2022 May 16;25(6):104409. doi: 10.1016/j.isci.2022.104409 (PMC9157191; doi:10.1016/j.isci.2022.104409)
Supplement: Document S1. Figures S1 and S2 and Tables S1–S4 [file mmc1.pdf]

iScience, Volume *25*

Supplemental information

The *ex vivo* pharmacology of HIV-1 antiretrovirals differs between macaques and humans

Carolina Herrera, Mackenzie L. Cottrell, John Prybylski, Angela D.M. Kashuba, Ronald S. Veazey, Javier García-Pérez, Natalia Olejniczak, Clare F. McCoy, Paul Ziprin, Nicola Richardson-Harman, José Alcami, Karl R. Malcolm, and Robin J. Shattock

Table S1. PK/PD parameters for each drug alone or in combination in human tissue explants for data shown in Figure 2.

Tissue	Dose[a]	TFV		TFVdp		MVC	
		C_{max} (ng/mg)	AUC_{3h-15d} (ng.d/mg)	C_{max} (fmol/mg)	AUC_{3h-15d} (fmol.d/mg)	C_{max} (ng/mg)	AUC_{3h-15d} (ng.d/mg)
Colorectal	Single High [ ]	2.92 (1.01)	6.62 (1.61)	22.41 (25.98)	98.95 (105.87)	3.15 (1.45)	6.96 (4.27)
	Single Low [ ]	1.32 (0.54)	2.47 (0.98)	8.36 (16.71)	35.29 (55.65)	0.22 (0.09)	1.38 (2.22)
	Comb. High [ ]	3.67 (0.47)	6.37 (1.00)	20.07 (23.22)	82.53 (86.63)	1.86 (0.32)	3.84 (0.87)
	Comb. Low [ ]	1.17 (0.38)	1.99 (0.64)	8.36 (16.71)	41.21 (71.45)	0.29 (0.07)	0.62 (0.30)
Ecto-cervical	Single High [ ]	13.59 (3.29)**	25.40 (7.03)**	N/D	N/D	2.61 (0.33)	4.95 (0.10)
	Single Low [ ]	2.03 (0.18)	4.05 (1.00)	N/D	N/D	0.56 (0.43)	1.23 (0.73)
	Comb. High [ ]	16.63 (0.54)****	30.61 (0.93)****	N/D	N/D	2.66 (0.32)*	5.55 (1.33)
	Comb. Low [ ]	2.38 (0.44)*	4.409 (0.61)*	N/D	N/D	0.59 (0.30)	1.22 (0.41)

[a] Composition of gels used: Single: Gel containing one drug, either TFV or MVC; Comb.: Gel containing a combination of TFV and MVC; High [ ]: TFV at 70 μM and MVC at 3.8 μM; Low [ ]: TFV at 7 μM and MVC at 0.38 μM.
The data are means (s.d.) derived from three independent experiments performed in duplicate.
N/D: Not Detected.

* Statistical significance towards ecto-cervical C_{max} or AUC_{3h-15d} in comparison with colorectal tissue explants was calculated using a two-tailed unpaired Student t test (* $P \leq 0.05$, ** $P \leq 0.01$, *** $P \leq 0.001$).

Table S2. PK/PD parameters for each drug alone or in combination in Rh. macaque tissue explants for data shown in Figure 3.

Tissue	Dose[a]	TFV		TFVdp		MVC	
		C_{max} (ng/mg)	AUC_{3h-15d} (ng.d/mg)	C_{max} (fmol/mg)	AUC_{3h-15d} (fmol.d/mg)	C_{max} (ng/mg)	AUC_{3h-15d} (ng.d/mg)
Colorectal	Single High [ ]	20.21 (44.26)	30.64 (66.33)	41.93 (23.03)	176.28 (76.82)	24.34 (54.96)	37.51 (82.50)
	Single Low [ ]	2.98 (5.51)	4.68 (8.16)	41.47 (15.49)	164.03 (54.69)	0.95 (1.49)	1.78 (2.24)
	Comb. High [ ]	44.16 (98.96)	66.86 (148.38)	29.12 (9.55)	148.40 (104.87)	35.40 (81.64)	54.31 (122.25)
	Comb. Low [ ]	9.19 (20.65)	13.99 (30.94)	24.43 (7.32)	107.96 (42.39)	2.79 (6.26)	4.72 (9.28)
Ecto-cervical	Single High [ ]	1.25 (0.42)	9.55 (3.38)	N/D	N/D	0.94 (0.50)	7.17 (3.80)
	Single Low [ ]	0.66 (0.25)	4.99 (1.91)	N/D	N/D	0.122 (0.05)	0.95 (0.31)
	Comb. High [ ]	2.46 (0.62)	18.55 (4.73)	N/D	N/D	1.19 (0.39)	9.03 (2.93)
	Comb. Low [ ]	1.86 (1.54)	14.11 (11.90)	N/D	N/D	0.64 (0.67)	4.93 (5.24)
Vaginal	Single High [ ]	1.37 (0.49)	10.25 (3.69)	17.89 (16.60)	134.18 (124.47)	1.44 (0.62)	10.88 (4.81)
	Single Low [ ]	0.54 (0.41)	4.05 (3.10)	6.16 (9.57)	46.22 (71.78)	0.19 (0.06)	1.44 (0.48)
	Comb. High [ ]	1.35 (0.69)	10.16 (5.15)	12.81 (11.06)	96.03 (82.94)	0.95 (0.25)	7.22 (1.98)
	Comb. Low [ ]	0.77 (0.70)	5.75 (5.30)	7.21 (12.68)	54.08 (95.07)	0.43 (0.42)	3.22 (3.18)

[a] Composition of gel used: Single: Gel containing one drug, either TFV or MVC; Comb.: Gel containing a combination of TFV and MVC; High [ ]: TFV at 70 μM and MVC at 3.8 μM; Low [ ]: TFV at 7 μM and MVC at 0.38 μM.

The data are means (s.d.) derived from independent experiments performed with six macaques in duplicate.

N/D: Not detected. N/A (Not applicable)

Due to sparse tissue only t_0 and day 15 samples were available, hence k_{el} could not be calculated.

Table S3. Area under the viral replication curve (p24/p27AUCτ) between days 3 and 15 in explants with or without drugs for experiments inhibitory potency summarized in Table 1.

Specie	Tissue	Virus	p24 or p27 AUC_{3-15} (μg^{x}d/ml)						
			Ctrl	TFV 70 μM	TFV 7 μM	MVC 3.8 μM	MVC 0.38 μM	TFV 70 μM + MVC 3.8 μM	TFV 7 μM + MVC 0.38 μM
Human	Cervical	HIV-1_{BaL}	2.79 ± 1.14	1.13 ± 0.10	2.55 ± 1.47	1.32 ± 0.36	2.61 ± 1.35	1.06 ± 0.12	2.38 ± 1.18
	Colorectal	HIV-1_{BaL}	4.82 ± 3.15	0.41 ± 0.17*	2.57 ± 1.81	0.59 ± 0.33*	2.68 ± 1.79	0.39 ± 0.15*	2.00 ± 1.40
		HIV-$1_{YU.2}$	29.46 ± 5.56	1.83 ± 0.18**	10.73 ± 5.56*	1.60 ± 0.57***	9.15 ± 5.28*	1.19 ± 0.36***	6.05 ± 1.42**
		HIV-$1_{YU.2\ K65R}$	0.84 ± 0.02	0.41 ± 0.03***	0.71 ± 0.06*	0.36 ± 0.05***	0.76 ± 0.10	0.33 ± 0.04***	0.66 ± 0.06**
		HIV-$1_{YU.2\ M184V}$	1.08 ± 0.36	0.52 ± 0.02	0.82 ± 0.05	0.26 ± 0.13*	0.70 ± 0.11	0.33 ± 0.08*	0.80 ± 0.15
		HIV-$1_{YU.2\ K65RM184V}$	2.15 ± 0.59	1.01 ± 0.11*	1.88 ± 0.07	0.82 ± 0.09*	1.60 ± 0.38	0.78 ± 0.19*	1.46 ± 0.18
Rhesus macaque	Cervical	SIV_{mac32H}	1.57 ± 0.57	1.25 ± 0.36	1.46 ± 0.52	1.27 ± 0.35	1.42 ± 0.44	1.20 ± 0.35	1.38 ± 0.46
		$SIV_{mac32H\ K65RM184V}$	1.67 ± 0.34	1.40 ± 0.30	1.65 ± 0.30	1.37 ± 0.30	1.62 ± 0.33	1.32 ± 0.22	1.54 ± 0.21
	Vaginal	SIV_{mac32H}	1.53 ± 0.30	1.27 ± 0.24	1.44 ± 0.28	1.26 ± 0.24	1.45 ± 0.28	1.20 ± 0.19*	1.39 ± 0.24
		RT-SHIV	1.74 ± 0.38	1.28 ± 0.27*	1.53 ± 0.29	1.27 ± 0.28*	1.54 ± 0.31	1.23 ± 0.28*	1.48 ± 0.32
		$SIV_{mac32H\ K65RM184V}$	1.74 ± 0.31	1.37 ± 0.30	1.61 ± 0.29	1.33 ± 0.30*	1.55 ± 0.28	1.32 ± 0.37	1.59 ± 0.36
	Colorectal	SIV_{mac32H}	8.27 ± 1.99	1.23 ± 0.14***	4.84 ± 1.77*	2.42 ± 0.53***	6.12 ± 1.35	1.23 ± 0.23***	3.43 ± 1.05***
		RT-SHIV	6.49 ± 4.04	1.45 ± 0.24*	4.27 ± 2.75	2.85 ± 2.01	5.25 ± 3.12	1.33 ± 0.23*	3.38 ± 2.14
		$SIV_{mac32H\ K65R}$	2.21 ± 0.61	1.43 ± 0.31	1.95 ± 0.54	1.29 ± 0.26	1.88 ± 0.71	1.28 ± 0.19	1.81 ± 0.49
		$SIV_{mac32H\ M184V}$	11.24 ± 5.27	4.05 ± 2.11	9.15 ± 5.00	4.68 ± 2.67	9.33 ± 4.75	2.86 ± 1.30	7.90 ± 4.28
		$SIV_{mac32H\ K65RM184V}$	1.85 ± 0.51	1.38 ± 0.27	1.70 ± 0.32	1.33 ± 0.22*	1.65 ± 0.32	1.31 ± 0.23*	1.62 ± 0.25

The data are means ± s.d. derived from three independent experiments performed in triplicate from human tissue and from independent experiments performed in duplicate with six macaques for wild type isolates and at least three animals for resistant isolates.

* Statistical significance towards p24 AUC_{3-15} or p27 AUC_{3-15} in untreated control conditions was calculated using a two-tailed unpaired Student t test (* $P \leq 0.05$, ** $P \leq 0.01$, *** $P \leq 0.001$).

Table S4. Non-linear models of TFV dose and p24/p27 response correlation of data shown in Figure 4.

Tissue	HIV-1(p24) / SIV(p27)	n	*p*	r^2	AIC[a] (3-param[b])	AIC (4-param[c])
Ecto-cervical	p24	8	0.006	0.87	58.7	77.2
	p27	12	0.015	0.61	78.9	81.2
Vaginal	p27	24	0.004	0.51	250.2	255.1
Colorectal	p24	16	N/A	N/A	253.5	344533.0
	p27	24	N/A	N/A	359.1	685151.4

n = number of paired measurements. The data are derived from at least three independent experiments performed in duplicate with gels containing one or two drugs.
p = probability of non-linear model fit.
r^2 = (1-) the sum of the squared distances from each fitted curved divided by the squared distances from a horizontal line.
[a]AIC = Akaike information criterion value. The 3-parameter non-linear model provided lower AIC values indicating a better fit than an alternative 4-parameter model.
[b]3-parameter model: $Log_{10}(p24/p27) = b+(a-b)/1+10^{((Log10TFV)-c)}$.
[c]4-parameter model: $Log_{10}(p24/p27) = b+(a-b)/1+10^{(((Log10TFV)-c)-e)}$.
N/A = Not Applicable.

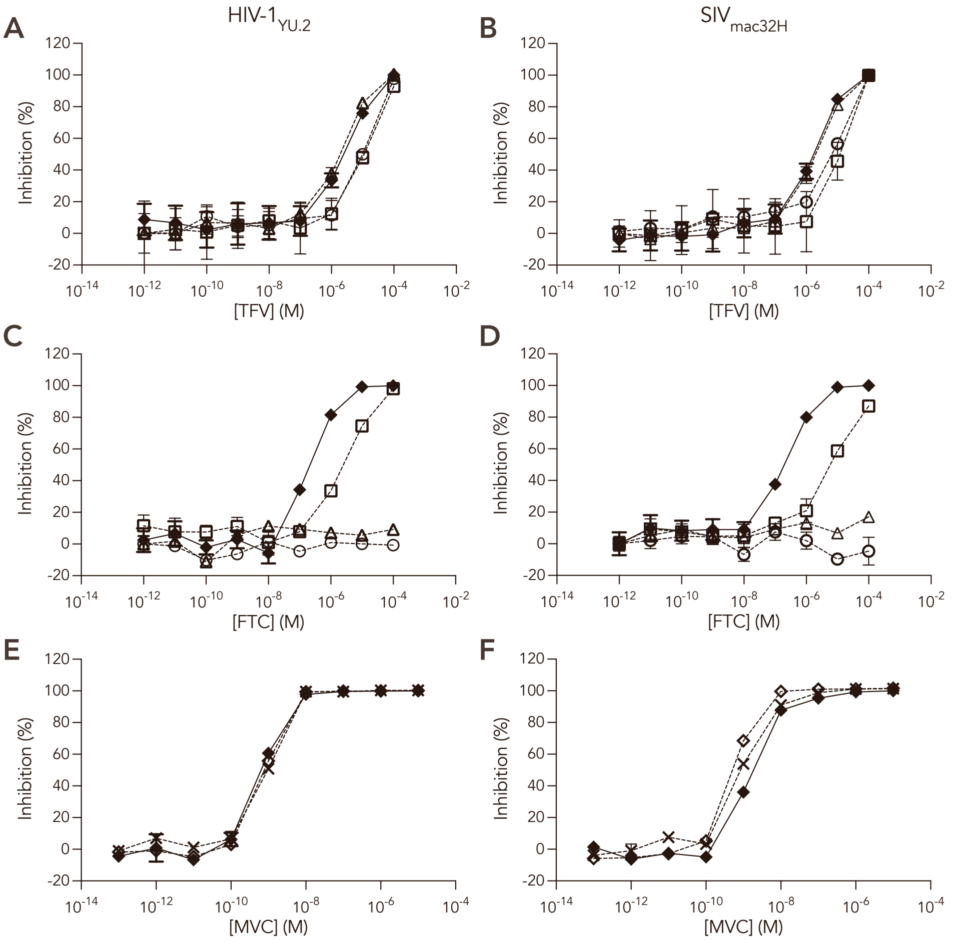

Figure S1. Titration of TFV, FTC and MVC inhibitory activity against wild type, RT- and Env-mutated HIV-1$_{cYU.2}$ and SIV$_{mac32H}$ in TZM-bl cells; related to clones described in STAR Methods. Resistance profiles for each clone generated was confirmed with TFV and emtricitabine (FTC) for the RT-mutants and with MVC for the Env-mutant. TZM-bl cells were treated for 1 h in the presence or absence of (A, B) TFV, (C, D) FTC or (E, F) MVC. Cells were then exposed to (A, C, E) HIV-1$_{cYU.2}$ or (B, D, F) SIV$_{mac32H}$ wild type (◆), RT-mutants K65R (☐), M184V (△), K65RM184V (○), or Env-variants L313T/I321V (◇), V314T/I321V (✕). Luciferase expression was determined after 48 h, and the extent of inhibition by each drug was calculated. The percentage of inhibition was normalized to the relative light units obtained for cells grown in the absence of virus (0% infectivity) and for cells infected with virus in the absence of drug (100% infectivity). Data are means ± SEM for three independent assays performed in triplicate.

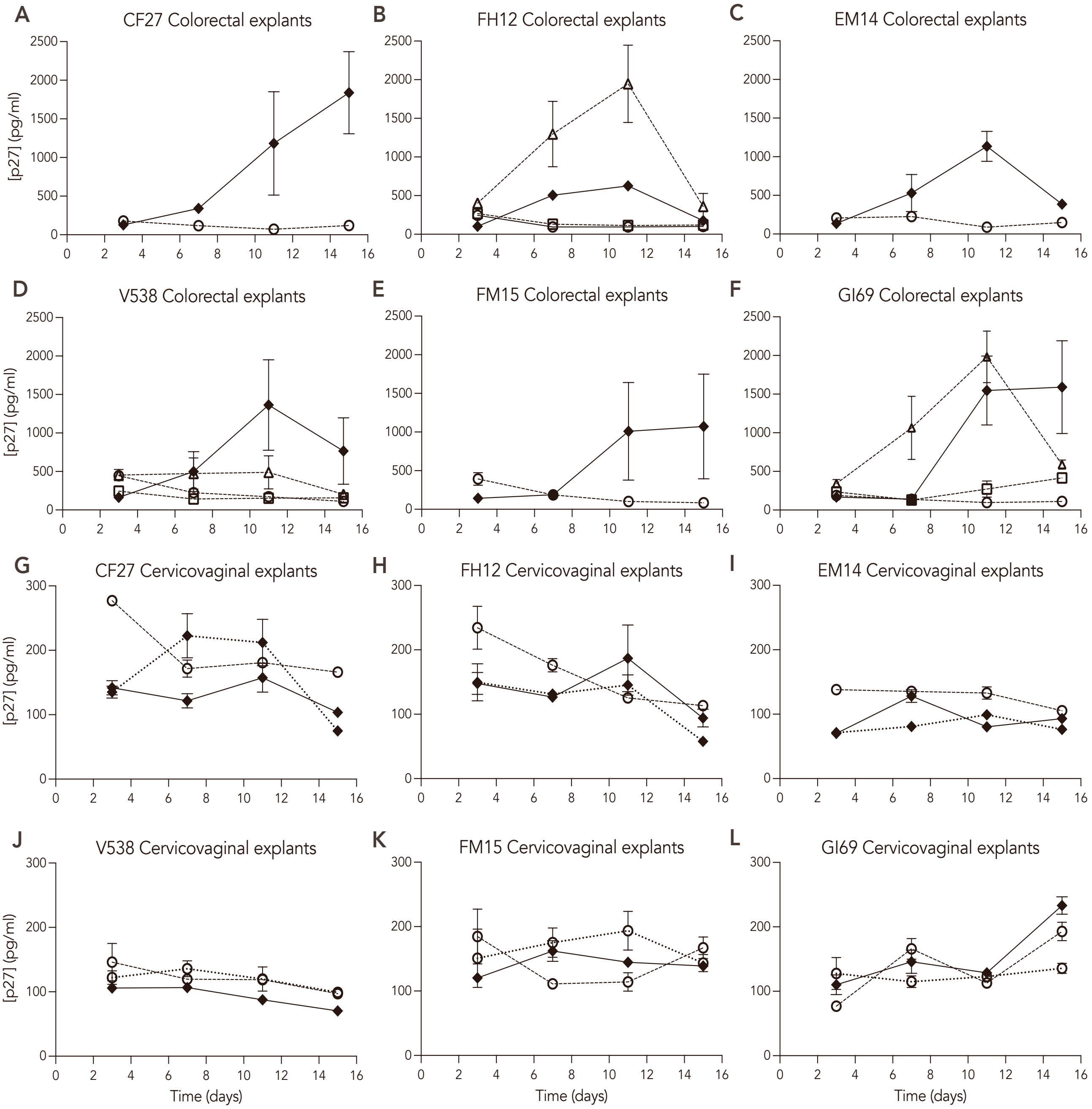

Figure S2. Replication kinetics of wild type and resistant SIV$_{mac32H}$ in NHP tissue explants in comparison to data shown in Figure 1. Colorectal, vaginal and ecto-cervical (dotted line) explants from Rhesus macaques were challenged with SIV$_{mac32H}$ (◆), SIV$_{mac32HK65R}$ (□), SIV$_{mac32HM184V}$ (△), SIV$_{mac32HK65RM184V}$ (○) for 2 h, washed and cultured for 15 days. Supernatants were harvested at different time points and p27 concentrations measured by ELISA. Data are means ± SEM from experiments performed in duplicate for each virus with mucosal tissue from each NHP.
